# Faculty perceptions of simulation programs in healthcare education

**DOI:** 10.5116/ijme.5641.0dc7

**Published:** 2015-11-22

**Authors:** Ana P. Quilici, Angélica M. Bicudo, Renan Gianotto-Oliveira, Sergio Timerman, Francisco Gutierrez, Karen C. Abrão

**Affiliations:** 1 School of Medicine, Anhembi Morumbi University, Sao Paulo, Brazil; 2 Pediatric Department, Medical Science College, UNICAMP, Campinas, Brazil; 3 Medicine and Health Sciences, Laureate Education, Baltimore, USA

**Keywords:** Undergraduate, simulation, education environment, communication skills, roles of teacher

## Abstract

**Objectives:**

To identify faculty perceptions of simulation insertion in the undergraduate program, considering the advantages and challenges posed by this resource.

**Methods:**

We conducted a qualitative study with intentional sampling according to pre-defined criteria, following a semi-structured outline regarding data saturation. We have interviewed 14 healthcare instructors from a teaching institution that employs simulation in its syllabi.

**Results:**

The majority of the faculty interviewed considered the use of scenario, followed by debriefing, as an excellent teaching tool. However, the faculty also noted a number of difficulties, such as the workload necessary to assemble the scenario, the correlation between scenario goals and the competences of the program, the time spent with the simulation, and the ratio of students to faculty members.

**Conclusions:**

Faculties consider simulation an effective tool in the healthcare program and maintain that the main obstacle faced by them is the logistical demand.

## Introduction

Healthcare education has undergone numerous paradigm shifts over the last few decades. Historically, a traditional teaching model was emphasized, providing a passive leaning experience. Today, the evolution of teaching methods has yielded a more student-centered learning process that departs from faculty-centered processes.^1^

Simulation is an example of the active methodology of teaching that allows for training in real conditions, with simulators and actors, in a controlled environment. These conditions result in the profound utilization of three important healthcare training elements: cognitive, psychomotor, and affective.^2^^-^^4^ Additionally, simulation also enables the repetition of procedures and reflection of conduct taken without patient exposure to possible human error related to the learning curve.^5^

The interest in using simulation has grown in the healthcare area. Considering its strengths, it has presented itself as a valuable tool in both training programs as well as formal education.^6^ Yet many universities and hospitals have expressed concern in building simulation centers to teach healthcare students. The infrastructure and technology are not enough to comply with the demands of teaching, and the healthcare faculty’s real challenge is to effectively utilize this tool. Ultimately, a major component of this challenge lies in attracting faculty members to apply this methodology.^7^

Even after much training in clinical simulation, difficulties still arise for some faculty in combining active and practical methodology, due to their approach to teaching and building critical thinking.^8^ Therefore, it is important to establish a deeper understanding of their approach and opinions.

Medical schools in Brazil and worldwide tend to present a more traditional profile, and a large part of faculty members demonstrate resistance to the introduction and application of new learning-teaching methodologies.^9^

Several factors can make the introduction of this kind of methodology difficult to incorporate into a syllabus. Among them we can cite the training skills, teaching programs, student profiles, motivation and faculty involvement, and material and human resource availability.^7^^,^^10^

The aim of this study is to identify the faculty perception of the advantages and challenges of simulation insertion, whereas a scenario is followed by debriefing, including its limitations and experience with the construction of the steps in the scenario as it pertains to healthcare educations programs.

## Methods

### Study design and participants

We conducted a qualitative study with intentional sample, according to predefined criteria, considering data saturation.^11^ Faculty from a private university in Sao Paulo, Brazil participated in the study. This institution has integrated clinical simulation in its program for all healthcare courses since 2008. The ratios of students to faculty in their trainings vary from 10 to 25 students per professor. The University Research Ethics Committee approved the project on December 3^rd^, 2013, publishing records in 477.231.

### Sampling and sample size

Faculty members were included in this study based on the following criteria: firstly, the use of debrief following training scenarios was required.  Secondly, the frequency of scenario followed by debriefing was required to occur at a minimum of once every academic quarter. Lastly, the selection of participants was based on a list provided by the university consisting 27 faculty members.

### Data-collection methods

For the interviews, we followed a semi-structured outline containing socio-demographic questions relevant to the aims of the study, which was pre-tested through 4 interviews with professors of physiotherapy. The data collection started at the beginning of December 2013, and went through to 30th of March, 2014. The interviews were performed by telephone.^12^^,^^13^ The interviewers did not have any connection to the study participants. They were skilled in performing interviews and were trained by the researcher to execute the task. 

The interviews were recorded, with the participants’ authorization. They were scheduled according to the professors’ conveniences and recorded directly in digital files with the assistance of the computer program “Call MonitorAdapt USB”. The time of the interviews ranged from 13 minutes to 27 minutes. The interviews were then transcribed, and the interviewers conferred the correspondent text contents, totalling 14 interviews. The collection stopped after the 14th interview due to an overabundance of information.

### Procedure

The study conducted 14 interviews, once it had reached the sample saturation. The concept of data saturation implies that the collection continues until information begins to repeat and they are adequate to the objectives of the study.^11^

Of the total collected, 9 participants were medical staff and 5 were nursing. Ten interviewees were female and 4 male. The age range varied from 37 to 63 years old. Teaching time varied from 3 to 20 years, and clinical simulation training with scenario followed by debriefing varied from 1 to 6 years. All participants had received training in assembling scenario followed by debriefing for a period between 8 hours minimum and 16 hours maximum. It is worth noting that such training hours were exclusively dedicated to the assembly of scenarios followed by debriefing.

### Data analysis

The data analysis was thematic and followed the methodology orientation of Patton.  Eleven categories of analysis from the significant issues identified in the interviews were established. In this study we will approach the views of faculties on the use of scenario followed by debriefing, separated into Advantages, Possible limitations and / or disadvantages, Experiences with the use of the scenario: Difficulties and challenges, and Experience with the construction of the three steps in the scenario (A. Determine the objectives; B. Construction of medical history; C. Planning).

## Results

### Opinion / advantages

All participants considered the use of scenario followed by debrief a great didactic tool. Some faculty emphasized that simulation is one of the most effective teaching tools and, therefore, it is essential that universities adopt it. 

“In my point of view simulation is one of the best tools I have ever seen in assisting my work and increasing the training for healthcare. I have now one way of teaching that differs from the one I was taught at the beginning of my career. So, today, medical education is much more advantageous due to this kind of resource, do you know what I mean?” (Faculty 7, physician, male, 48 yr)  

When asked about possible advantages in using simulation, nearly half of the interviewees noted that the use of scenario followed by debrief allowed the student to make a mistake in a controlled way, which contributes to minimizing possible future mistakes while at the same time granting greater confidence to the student.

“I believe that it minimizes the mistakes when in contact with the patients, generating more confidence and more psychomotor skills, clinical and logical thinking relate to the cares that will be taken towards the patient.”   (Faculty 13, nurse, female, 43 yr)

One participant noted that despite the differences between real medical treatments and simulation, scenario training still at least supplies the students with a dynamic experience of a treatment. She did emphasize, however, the importance of training in hospitals and emergency rooms for student learning.

“They have already had a contact at least with the dynamic of the process, one that at the scenario, you know? But, I guess it is good. However, it doesn´t exclude the training activities the must have together with hospitals and emergency rooms!” (Faculty 2, physician, female, 46 yr)

Also, in respect to the advantages of using scenario followed by debrief, it was observed that the tool allows the student to adapt to working in teams, which improves communication between professor and student, making them closer. It was also expressed that realistic simulation is more exciting, causing the student to be more focused in class.

“I think that it is an opportunity that we have in getting the student to see what he is doing wrong or if he is doing right. I think that, practically, in the student acting, he can learn much more than just listening how it must be done.” (Faculty 4, physician, female, 40 yr)

“I think that scenario followed by debriefing is very good because it keeps the learning. The big differential is that the debriefing is a stimulus to the students thinking and it makes them study.”  (Faculty 6, nurse, female, 56 yr)

The best advantage of the clinical simulation is that it assists students in drawing their own conclusions.

“The faculty places himself at the same level of students, not imposing any conclusion. Therefore, students accept more. They arrive to conclusions by themselves. So, they accept better. They assimilate better.” (Faculty 3, physician, male, 32 yr)

### Possible limitations/disadvantages

There were some statements regarding limitations in the use of scenario followed by debriefing. A faculty argued that while it is a good teaching tool, its use applies to specific situations.

“I think it is the main point, I mean, it is a tool, not a salvation. It is not a base for education. – Well! I will only do it if it is with the realistic simulation. No! Then, that is the reason that for me it is clear, I see it as a tool, a very good tool, as I said, it is not the only one.” (Faculty 3, physician, male, 32 yr)

Furthermore, the amount of workload and time available to develop simulations are also perceived as limiting factors.

“The simulation, although it is able to convey various kinds of information, is not always homogeneous. It has to be revised and reissued many times. Maybe we cannot maintain a theme to only one simulation. I think we need more time to simulation.” (Faculty 7, physician, male, 47 yr).

### Experiences with the use of the scenario: difficulties and challenges

When asked about their experience with the use of scenario followed by debriefing, the majority of participants gave answers related to students and to the time demanded by the tool’s use. They stated that students demonstrate resistance at first to simulation training, which could be a challenge for faculty when faced with students often passive or even constrained by having to expose themselves.

“... the challenge is that some students don’t like at first to expose themselves  and secondly they don’t like the mannequins, which present limitations as compared to reality.” (Faculty 10, physician, female, 37 yr)

It was noted that the number of students might present difficulties in the planning the scenario or even in its realization.

“And sometimes the number of students is big and you need to approach very specific objectives and with a large number of students maybe you can’t reach the whole group.” (Faculty 13, nurse, female, 43 yr)

Half of the participants experienced difficulties with the amount of time that the use of scenario followed by debrief demands from faculty.

“The logistics of the planning, it becomes a little bit, at the beginning mainly, it is a little bit more difficult. Over time, when you are doing your training, it becomes each time easier? So, I don’t see too many difficulties in planning. I believe that it really is a little more difficult at the beginning for you to connect all the situations that you will have to expose to your students. You will have to connect all the moments that you will engage your students, all the topics you need to approach, all the maneuvers you need to make and ask them to perform.” (Faculty 7, physician, male, 48 yr)

“...Another challenge is the time for preparation, because it demands preparation. You write a study case, state the objectives, work, focus on what is important… not allowing to go out of the track or to mix up too many things. We also, sometimes, feel insecure – will we accomplish it? Will we make it in due time? inIn class time? Sometimes the class time is not good enough, so it is a challenge that the faculty has to overcome.” (Faculty 13, nurse, female, 43 yr)

One participant expressed that a primary challenge was achieving the clarity of objectives necessary to be reached in a class:

“The main challenge is to have very clear which objectives you want students to achieve, from this then to come up with a clinical situation. In order to have sense in what we are going to do. So…set up the class objective and the ideal relation of the case study with what will happen in reality.”   (Faculty 8, physician, male, 43 yr)

### Experience with the construction of the three steps in the scenario (A. Determine the objectives, B. Construction of medical history; C. Planning)

Altogether, the participants mentioned that they followed the three steps in assembling the scenario. Some, however, stated that they did not recall the three steps, and rather referenced information at the time of constructing the scenario.

“According to my steps, I build them one at a time. I draw up a mental outline of what I want to demonstrate to students. From that, I start to build the steps. I figure out a kind of situation, a simulated situation, when I want to show a kind of clinical case, then I see the kind of patient, after what he presents, how students will approach him, after the decisions that the students must make.” (Faculty 7, physician, male, 48 yr)

Although some participants reported that they do in fact follow the steps and do not have difficulty in defining objectives, their interviews suggested that they do not have a correct understanding regarding the construction of an objective of the scenario.

“I follow exactly the steps. Then, at first I define what the students must have as an objective. Let’s take as an example; to learn how to intubate a patient, the objective is this, ok? How am I going to do it? Making up a clinical case in which the patient needs an intubation and from that I discuss it with them.” (Faculty 1, physician, male, 63 yr)

“For example, how to perform a fundoscopy, that has a complete examination, that allows to see an approach a diagnosis. Thus, we have experience in it. So, I don’t have any difficulties in doing it.” (Faculty 4, physician, female, 40 yr)

For all interviewees, the construction of the scenario is something pleasant. It is, however, a task that demands significant consideration. A faculty reports his enjoyment in observing the final results with the students.

“It is a pleasure. But at the same time, it is an intense mental activity, to make each one of the steps, to a specific scenario, to a specific content, to a specific moment of the student in the course. So, I get myself many times revising the definition of nursing, what are the competences of the nurse at such situation. I end up noticing limitations of the job, in the major possibilities of the job. Thus, it is an exercise of deep thinking, of research, of experience, of discussion…” (Faculty 9, nurse, female, 51 yr)

No participant reported difficulties regarding the availability of resources to build scenarios.

## Discussion

Studies increasingly show that with the integration of simulation in medical programs, the adherence of the faculty becomes fundamental.^14^ One of the elements that aids in faculty adherence is the understanding that these resources can improve medical trainings.^9^ There are several studies presenting the advantages of using clinical simulation, which include the safety of patients^15^ from the possibility of mistakes, the repetition of actions many times without harm, and the possibility of training real patients that are not always available in clinical training.^10^^,^^16^^,^^17^ The combination of these advantages turn simulation into a precious tool, when well applied.^9^^,^^18^

The development and training of skills such as communication, leadership, and teamwork is essential to healthcare education in general. Yet the means of developing these competences continue to be largely discussed. The use of scenarios followed by debriefing has in a large part proven to be an excellent tool for student development.^19^^-^^21^ This is evident by the fact that all interviewees considered the use of scenario followed by debrief a great didactic tool.

The learning process through simulated situations has proven to be an effective and useful method to evaluate performance and clinical skills, because it allows the control of external factors, the standardization of problems presented by patients, and the ability to provide positive feedback to students, increasing their self-knowledge and confidence.^22^ It also provides the opportunity for clinical learning to be centered on the patient, guaranteeing better interpersonal relationships, resolution of problems, and analysis and synthesis of clinical information, even without the use of real patients.^22^^,^^23^

Some studies, such as Ropé^24^ and Tanguy^25^, discuss the difficulty faculty face in identifying competences, as well as defining the universes and environments in which they are used. From such considerations, one must note that conceptual uncertainty is the main issue faced in statements of the interviewed faculty, as they struggle to define for sure the meaning of the concept of competence.^26^^,^^24^ In this study, the main difficulties and limitations stated by participants was the amount of workload and time available to develop simulation followed by debrief.

Ten Cate^27^ suggested that medical faculty have difficulty understanding the concepts underlying the curriculum and placing them into practice. These facts pose the question: is our faculty truly skilled enough to understand the curriculum concepts based in competences and practice them? An important issue to the success of this model surrounds faculty training; faculty must not only understand the simulation, but more importantly, they must also understand the curriculum model. The comprehension of the curriculum based on or guided by competence helps explain what actually needs to be developed, whereas the idea is not to transfer the contents of something in a scenario, but rather to practice in a controlled environment of determined clinical situations to develop such competences.^24^^,^^26^

The ratio between student and faculty during the scenario and debrief is a highly important issue stated by faculty members. Undoubtedly, in large groups with just one instructor, it may be difficult to perform and apply the scenario and the debriefing, and thus this is a question to consider before inserting clinical simulation into the curriculum. There is not yet published literature on an established policy regarding student/faculty ratios for simulation. Articles reporting efficacy of debriefing in their studies use a relation of 1 facilitator to an average of 6 to 10 participants.^28^^,^^29^ However, Barbara Steinwachs^32^, in her article “How to Facilitate a Debriefing“, states it is possible to perform a scenario and debriefing with as many as 20 to 25 participants. In this study, half of the participants’ experience difficulties with regards to the time consumed by scenario followed by debriefing, and stated that students can offer resistance at first to the simulation training. This proves to be a challenge for faculty when faced with students often passive or even constrained by having to expose themselves.

An interesting question is the difference between objectives of scenario and the skill competence. The performance of a fundoscopy or to learn how to intubate a patient, for example, must not be objectives of a scenario, but rather a skill trained. The decision-making in performing a rapid sequence airway or a fundoscopy in a determined clinical situation consists of the objective of a scenario. Such statements suggest that even for experienced faculty members, the struggle to define scenario objectives is still a challenge to be faced. The anxiety in using the tool can also be a challenge to be overcome, but as faculty gains experience, these challenges are easily overcome. This corroborates what is stated regarding the participants’ difficulties in understanding competences and not contents.^26^  

The work and time needed to prepare one class using simulation is, no doubt, very much involved and very challenging compared to an expositive one. The scenario construction implies assuring clarity regarding the competences that you want to reach in order to determine the objective. It is necessary to develop the entire evolution of the scenario, test rigorously before applying it, and consider heavily the manner in which to conduct the debriefing.^30^^,^^31^ Therefore, it is necessary to prepare amply for the class, thus making possible the use of simulation. The clarity in determining the objectives of the scenario is directly related to the clarity of the competence that you want to foster in the scenario.^31^ While they may not have memorized the three steps of construction, there were faculty that described a logical sequence to do so. All participants stated that the construction of the scenario is something pleasant and that they do not struggle with that component.

Despite assuming that interview data typically has higher reliability and validity than survey data,^32^ this study did have some limitations. The study was conducted on faculty at only one university, thus the findings are not necessarily generalized to any other institution. Another limitation is the number of participants that sometimes cannot produce a truly representative general opinion, but subject one. Consequently, further studies of the perceptions of simulation as a summative assessment, with immediate feedback, would be useful in building our understanding of faculty engagement with realistic simulation, including its impact, value and sustainability, as well as learning, development and confidence.

## Conclusions

The information analysis of the statements allows us to conclude that: faculties consider simulation a useful tool in the healthcare program and the main difficulties reported by them are logistics. Therefore, there are logistical needs to be addressed and one of these points is to revise the ratio of students to faculty members in trainings involving simulation, so they can effectively apply the proposed methodology. Although, the study was conducted with participants from one university, the understanding of how teachers think about teaching with simulation, including how we can understand real difficulties, can help other universities strengthen their training programs and integration of simulation into their curriculums.

### Conflict of Interest

The authors declare that they have no conflict of interest.
